# Macro-Scale Strength and Microstructure of ZrW_2_O_8_ Cementitious Composites with Tunable Low Thermal Expansion

**DOI:** 10.3390/ma11050748

**Published:** 2018-05-07

**Authors:** Jianshu Ouyang, Yangbo Li, Bo Chen, Dahai Huang

**Affiliations:** 1 School of Transportation Science and Engineering, Beihang University, Beijing 100191, China; auyeung@buaa.edu.cn (J.O.); huangdahai@buaa.edu.cn (D.H.); 2 College of Hydraulic and Environmental Engineering, China Three Gorges University, Yichang 443002, China; 15672487483@163.com

**Keywords:** ZrW_2_O_8_, cementitious composites, low thermal expansion

## Abstract

Concretes with engineered thermal expansion coefficients, capable of avoiding failure or irreversible destruction of structures or devices, are important for civil engineering applications, such as dams, bridges, and buildings. In natural materials, thermal expansion usually cannot be easily regulated and an extremely low thermal expansion coefficient (TEC) is still uncommon. Here we propose a novel cementitious composite, doped with ZrW_2_O_8_, showing a wide range of tunable thermal expansion coefficients, from 8.65 × 10^−6^ °C^−1^ to 2.48 × 10^−6^ °C^−1^. Macro-scale experiments are implemented to quantify the evolution of the thermal expansion coefficients, compressive and flexural strength over a wide range of temperature. Scanning Electron Microscope (SEM) imaging was conducted to quantify the specimens’ microstructural characteristics including pores ratio and size. It is shown that the TEC of the proposed composites depends on the proportion of ZrW_2_O_8_ and the ambient curing temperature. Macro-scale experimental results and microstructures have a good agreement. The TEC and strength gradually decrease as ZrW_2_O_8_ increases from 0% to 20%, subsequently fluctuates until 60%. The findings reported here provide a new routine to design cementitious composites with tunable thermal expansion for a wide range of engineering applications.

## 1. Introduction

Cementitious Composites (CCs) are facile, economical structural materials, which are most widely applied in civil engineering [1]. But CCs have such innate shortcomings as brittleness, poor tensile strength, and poor thermal conductivity. In order to enhance structural integrity, researchers and engineers have usually focused on tuning their stiffness, strength, fracture toughness [2], self-healing capability [3,4], ductility [5,6], hydration [7,8] and shrinkage [9,10] through filling the cement matrix with micro-/nano- fibers [11], graphene [12,13], carbon nanotubes [14,15,16,17], silica fume [18,19], magnesium oxide (MgO) [20,21], plasticizer [22,23], hardening accelerator [24], and fly ash [25,26]. Among various loading conditions that often occur in concrete structures, the thermal load is a ubiquitous one, especially in massive concrete structures, causing thermal cracks or fractures, which are always the severest challenge for integrity and perfection of concrete structures. Thermal-induced cracks are often found in massive structures, for example, dams, bridges and buildings like China Three Gorges Dam, the Cathedral of Our Lady of the Angels in California, USA, and Oddesund bridge in Denmark [1]. Until now, the problem of how to improve concrete’s capability of resisting thermal load has not been effectively solved. Conventional approaches include enhancing concrete’s strength and lessening thermal stress. Enhancing concrete strength is implemented by increasing the quantity of cement per cubic meter, which usually accompanies increasing the Young’s modulus of the concrete and hydration heat of the concrete and causes easier failure due to temperature amplitude. Lessening thermal stress is realized via controlling the initial temperature of concrete, hydration heat by low-heat substitution of cement, embedded cooling pipes and slicing unity into sections, which scarifies the unity of massive concrete structure and simultanously results in higher costs and a longer construction process [27,28], since concrete is a poor conductor of heat. The application of 3D (three dimensional) printing technology in concrete fabrication [29,30,31], due to rapid prototyping, from fluidic to well-hydrated in a short time, without a mold, can lead to severer thermal stress and distortion in 3D-printed cementitious composites, analogous to the cementitious mortar.

According to classical thermo-mechanical theory, the upper limit of thermal stress is given as σ=EαΔT, where *E* is the Young’s Modulus, *α* the thermal expansion coefficient (TEC), and Δ*T* temperature increment. Therefore, reducing concrete’s TEC is an alternative and soluble approach that might lessen thermal stress. Recently, many artificially synthesized materials with negative thermal expansion (NTE) spring up, for instance, Zirconium tungstate (ZrW_2_O_8_) and zirconium pyrovanadate (ZrV_2_O_7_) [32,33,34,35], which provide opportunities to decrease the TEC of synthesized composites. Metallic [36,37], metallic oxide [38,39], asphalt mastic [33] or polymeric [40] composites have realized the NTE effect by mixed with ZrW_2_O_8_. Therefore, compared with conventional approaches, tuning TEC via doping with ZrW_2_O_8_ makes CCs insensitive to temperature variation to avoid cracks and ensure structural integrity without such side effects as extra elastic modulus increase, extra hydration heat, and extra costs. ZrW_2_O_8_ as filler in cement matrix was preliminarily explored in random synthesis [41]. But the feasibility of applying ZrW_2_O_8_ cementitious composites (ZCCs) in concrete is determined by their strength, stiffness, and toughness. Whether the NTE property of ZrW_2_O_8_ functions in the concrete structures’ service environment is also rarely evaluated in prior research. Additionally, no one underscores the TEC of the concrete to lessen thermal stress.

Here, inspired by NTE synthesized composites, we present a new type of cementitious composite with tunable low thermal expansion, by mixing them with differently proportional ZrW_2_O_8_. To assess their feasibility in engineering applications, the macro-scale thermo-mechanical experiments are performed to measure their TEC, compressive strength and flexural strength. The dependence of low thermal expansion and flexural and compressive strength on ZrW_2_O_8_’s percentage is also investigated by comparing the experimental results. Subsequently, the Scanning Electron Microscope (SEM) imaging is conducted to observe samples’ microstructures including pore ratio and sizes, and to reveal the thermal deformation mechanism in the micro-scale. The feasibility and applicability of ZCCs in engineering applications are also discussed to ascertain the necessity of further researches on ZCCs.

## 2. Materials and Testing Methods

### 2.1. Materials

Standard cement mortars with 40 mm × 40 mm × 160 mm, mixed with different proportions of ZrW_2_O_8_ were made according to *Method of testing cement- Determination of strength* (GB-T 17671-1999) [42]. Experiments were conducted to test the coefficient of thermal expansion and the compressive and flexural strength. To explore ZrW_2_O_8_’s contribution to reducing the TEC of cementitious composites, seven groups of specimens were designed by the weight of ZrW_2_O_8_ with the weight ZrW_2_O_8_/Cement Ratio (Z/C) = 0%, 10%, 20%, 30%, 40%, 50%, and 60%, whereas per group compromises three same specimens.

**Figure 1:**
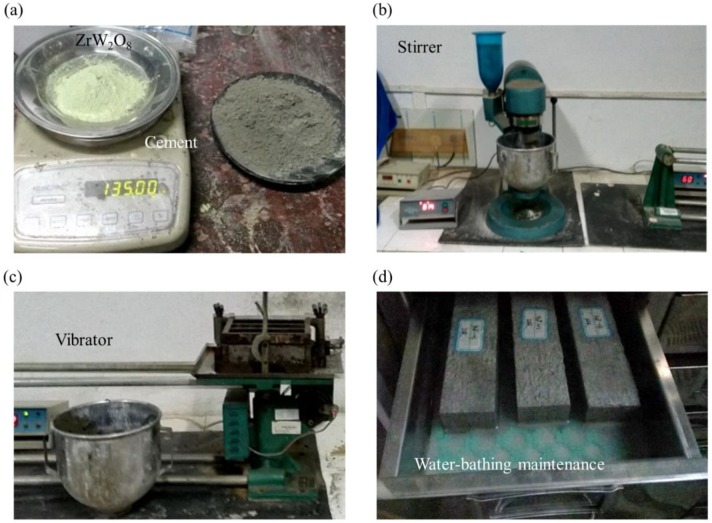
Fabrication of cementitious composites including synthesis, molding, hydration, solidification, and maintenance. (**a**) ZrW_2_O_8_ and cement are the main ingredients of cementitious composites; (**b**) The stirrer is used to blend uniformly all the ingredients; (**c**) The vibrator is employed to exclude pores inside and densify composites; (**d**) Specimens are placed, hydrate and solidify in a water-bathing maintenance environment.

Ordinary Portland cement P.O42.5 (SanXia brand) used in this experiment as shown in Figure 1a consists of such chemical compositions as 3CaO·SiO_2_ (C_3_S), 2 CaO·SiO_2_ (C_3_S), 3CaO·Al_2_O_3_ (C_3_A) and 4CaO·Al_2_O_3_·Fe_2_O_3_ (C_4_AF), ranging 48%, 26%,11%, and 16%, respectively. Chinese ISO standard sand is adopted, whose grain size ranges from 0.08 mm to 2 mm. ZrW_2_O_8_, provided by Tiegao international trade limited company of Shanghai as shown in Figure 1a, is a gray powder composed of angular particles typically in the size range from 4 µm to 7 µm. Drinking water is used to stir smooth.

### 2.2. Fabrication of Specimens

Fabrication of specimens includes such main stages as synthesis, molding, hydration, solidification, and maintenance, as shown in Figure 1b–d. The composites consist of the cement, standard sand, water, and different proportions of ZrW_2_O_8_ as shown in Table 1. In the first stage, in order to evenly disperse ingredients, the synthesis has to obey the following procedures: the water, the cement and ZrW_2_O_8_ are poured first into the pot of the planet-like stirrer (Figure 1b) ready to be stirred for 30 s at a low speed. After that, standard sand is mixed into the composites with the high speed of stirring 30 s. Before completing the synthesis, the stirrer pauses for 90 s and then keeps going for 60 s. To avoid over-vibration or less-vibration of the mortar specimens, time deviation every step should be controlled less than one second.

Later, the composites are cast into 40 mm × 40 mm × 160 mm molds. To make specimens denser, the three linked molds with fluidic specimens should be vibrated on the specific machine as shown in Figure 1c. After totally compacted, the specimens are maintained in the constant temperature and moisture environment of 20 °C and 80%. This stage lasts for 24 h until specimens are removed from the molds. Eventually, they are placed in a water-bathing environment to hydrate and solidify for 28 days as shown in Figure 1d.

### 2.3. Testing

To clarify ZrW_2_O_8_’s role on the improvement of thermo-mechanical properties, the TEC, compressive strength, and flexural strength of differently proportional ZrW_2_O_8_ specimens was investigated in sequence. All the specimens experienced same maintaining and thermally and mechanically loading process. All the specimens’ fabrication and the testing methods abide by *Method of testing cements-Determination of strength* (GB-T 17671-1999).

#### 2.3.1. Testing the thermal expansion coefficient (TEC)

**Figure 2:**
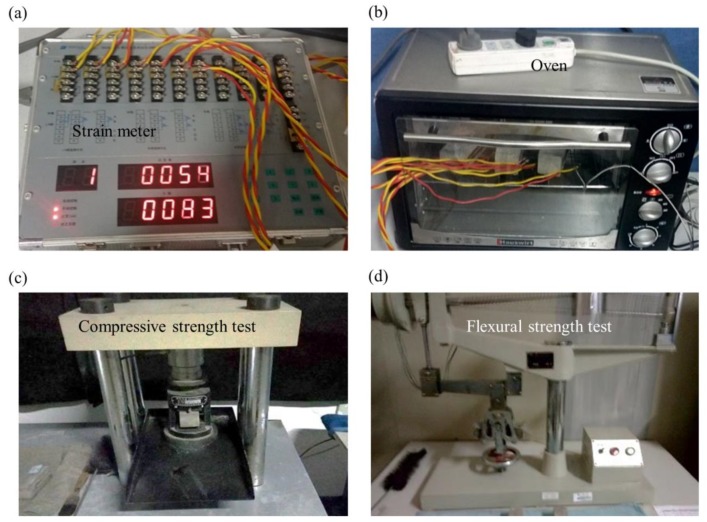
Experiments of thermal strain, compressive and flexural strength testing. (**a**) The strain meter aims to capture the thermal strain during the heating process; (**b**) The thermal load is realized by increasing the temperature in the oven; (**c**) The compressive testing machine; and (**d**) the flexural testing machine are applied to test the strength of specimens.

The TEC of specimens is measured by a strain meter (DH3815, Donghua Co., Ltd., Taizhou, China, Figure 2a) within a glass-window thermal oven with a controlled temperature as shown in Figure 2b. The temperature was gradually increased from room temperature (20 °C) to 120 °C and held for 30 min for each step to achieve a homogenous temperature distribution. Two strain gauges pasted by epoxy glue on literally opposite surfaces on the specific specimen are used to capture the thermal strains with the temperature increase. To ensure the strain gauges to represent the real thermal strain of specimens, we employ an extra constant strain gauge as the compensation, paste tightly two measured gauges connected to the strain meter, and take the average value of both. Before performing thermal load, we need to dry specimens more than 24 h and calibrate strain gauges. During the testing process, we read the strain value after calm indication on the meter.

#### 2.3.2. Flexural Strength Determination

The flexural strength is tested by double-lever electric testing machine (KZJ-5, Xidong CO., Ltd., Wuxi, China) as tri-point bending style as shown in Figure 2c. The machine loads as the velocity of 50 N/s on the specimen and records the force, *F_f_*, at the moment the specimen is destroyed. The flexural strength is given by ${\left[\sigma \right]}_{f}=\frac{1.5F_{f}L}{b^{3}}$, where *L* is the distance between the supports, and *b* the width of cross section area.

#### 2.3.3. Compressive Strength Testing

After flexural strength determination, every specimen is split into two halves, which are continuously used as samples to test the compressive strength on the testing machine (YAW-300, Zhongke Co., Ltd., Wuxi, China) as shown in Figure 2d. The machine loads at the velocity of 2.4 KN/s until the sample is destroyed.

### 2.4. Scanning Electron Microscope (SEM) Imaging

After performing macro-scale mechanical testing, the SEM imaging is conducted to observe specimens’ pores ratio and size via SEM instrument (JSM-7500SEM, JEOL, Tokyo, Japan) and Analysis Software (Image Pro-Plus, Media Cybernetics, Inc., Rockville, MD, USA). We take some minor pieces of fragments from destroyed cross area on the specimens as imaging samples.

### 2.5. Theoretical Prediction of TEC

We adopt weighted average method in elastic modulus to prediction the TEC of ZCCs. The theoretical model is conducted as

(1) $$\alpha =\frac{\alpha_{p}E_{p}V_{p}+\alpha_{s}E_{s}V_{s}+\alpha_{g}E_{g}V_{g}}{E_{p}V_{p}+E_{s}V_{s}+E_{g}V_{g}}$$

where $\alpha_{p}，\alpha_{s}，\alpha_{g}$ are the TECs of cement stone, sand and ZrW_2_O_8_, respectively, and $E_{p}，E_{s}，E_{g}$ the elastic modulus of cement stone, sand and ZrW_2_O_8_, and $V_{p}，V_{s}，V_{g}$ the volume fraction of cement stone, sand and ZrW_2_O_8_. Here $V_{p}+V_{s}+V_{g}=1$. The TEC of cement stone is 15–20 × 10^−6^ °C^−1^, TEC of sand 12 × 10^−6^ °C^−1^, and TEC of ZrW_2_O_8_ −8.7 × 10^−6^ °C^−1^.

## 3. Results and Discussion

### 3.1. Macro-Thermo-Mechanical Properties

**Figure 3:**
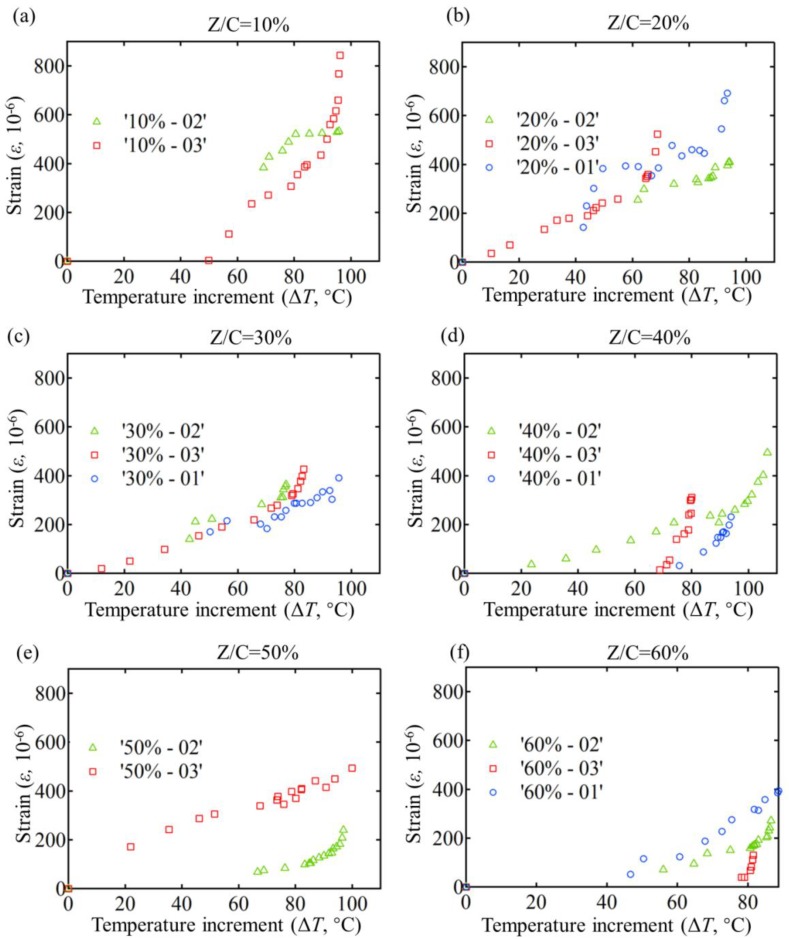
The thermal response of different proportional ZrW_2_O_8_ cementitious composites specimens with Z/C = (**a**) 10%, (**b**) 20%, (**c**) 30%, (**d**) 40%, (**e**) 50%, and (**f**) 60%. (The titles in the legends in Figure 3 mean “ZrW2O8/Cement in weight—the number of the specimens with the same mix design”).

**Figure 4:**
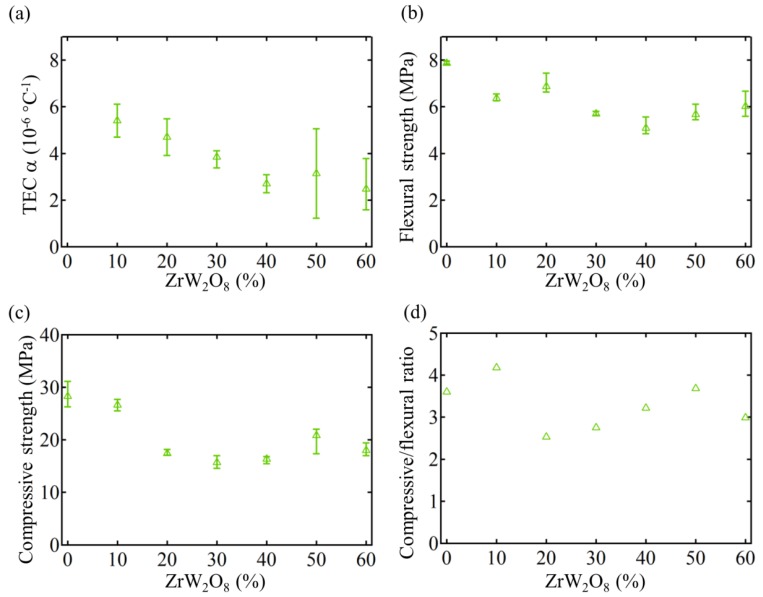
The change of (**a**) thermal expansion coefficient (TEC), (**b**) flexural strength, (**c**) compressive strength and (**d**) compression/flexure ratio with the increase of ZrW_2_O_8_ proportions.

Figure 3 reports the changes of thermal strains with the increase of temperature from room temperature (20 °C) to 120 °C among different proportional ZrW_2_O_8_ cementitious composite specimens. Figure 4 shows the evolutions of TEC, flexural strength, compressive strength and compression/flexure ratio with the increase of ZrW_2_O_8_. The standard cement mortar’s compressive strength at 28 d age is 28 MPa, flexural strength is 7.8 MPa, which indicates that the experiments are reliable. According to theoretical prediction mentioned above, we calculated the TEC of 0% ZCC, *α*_0%_ = 8.93 × 10^−6^ °C^−1^, and the TEC of 30% ZCC *α*_30%_ = 4.08 × 10^−6^ °C^−1^, which is close to the experimental results and further verifies the reliability of our experiments. 

Comparing thermal strains in Figure 3a–i, we observe that in general, as the proportion of ZrW_2_O_8_ increases, the maximum thermal strain gradually falls from approximately 800 to 300 × 10^−6^, which is 62.5% of non-ZrW_2_O_8_ CCs. But, at a relatively low level of temperature increment, less than 80 °C, thermal responses of all ZrW_2_O_8_ cementitious composites remain steady. When temperature moderately rises to 80 °C or higher, the thermal strains of all the specimens climb considerably high values, which vary with the percentage of ZrW_2_O_8_. It manifests that sand and ZrW_2_O_8_ play the same roles in influencing the TEC of the composite when the ambient temperature is less than 80 °C. After that, when ambient temperature is larger than 80 °C, the negative thermal expansion (NTE) property of ZrW_2_O_8_ takes effect, pulling back positive thermal expansion from the sand. Hence, the TEC of ZCCs is a function of ambient temperature rather than a constant value. In overall, the TEC of ZCCs reduces 65% with ZrW_2_O_8_ increase, compared with non-ZrW_2_O_8_ CCs. However, in civil engineering applications, such as dams, bridges, buildings and so on, most service environments or thermal loads are lower than 80 °C, which indicates that ZCC is a possible candidate for reducing thermal stress or distortion.

Furthermore, the strength of the NTE materials is crucial for potential applications. To study the mechanical properties, especially the strength of the proposed composites, uniaxial compression and 3-point flexure are carried out according to the standard testing method. The evolution of TECs, flexural and compressive strength, as well as compression/flexure ratio with ZrW2O8’s proportion increase in Figure 4, show that the TEC and strength gradually decrease as ZrW_2_O_8_ increases from 0% to 20%, and subsequently fluctuates until 60%. ZrW_2_O_8_ used in our experiment is a gray powder composed of angular particles typically in the size range from 4 to 7 µm, while Chinese ISO standard sand adopted ranges from 80 µm to 2000 µm in grain size. Mixture of sand and cement causes capillary cavities or voids between grains and cement particles. And ZrW_2_O_8_ powder will likely fill capillary cavities or voids when it is doped. That is why the ratios and sizes of porosities decrease with increase of ZrW_2_O_8_. In general, the strength of cement mortar decreases with the increase of percentage of small-size particles, because small particles cause weaker binding in hydrated mixtures.

### 3.2. SEM Analysis

**Figure 5:**
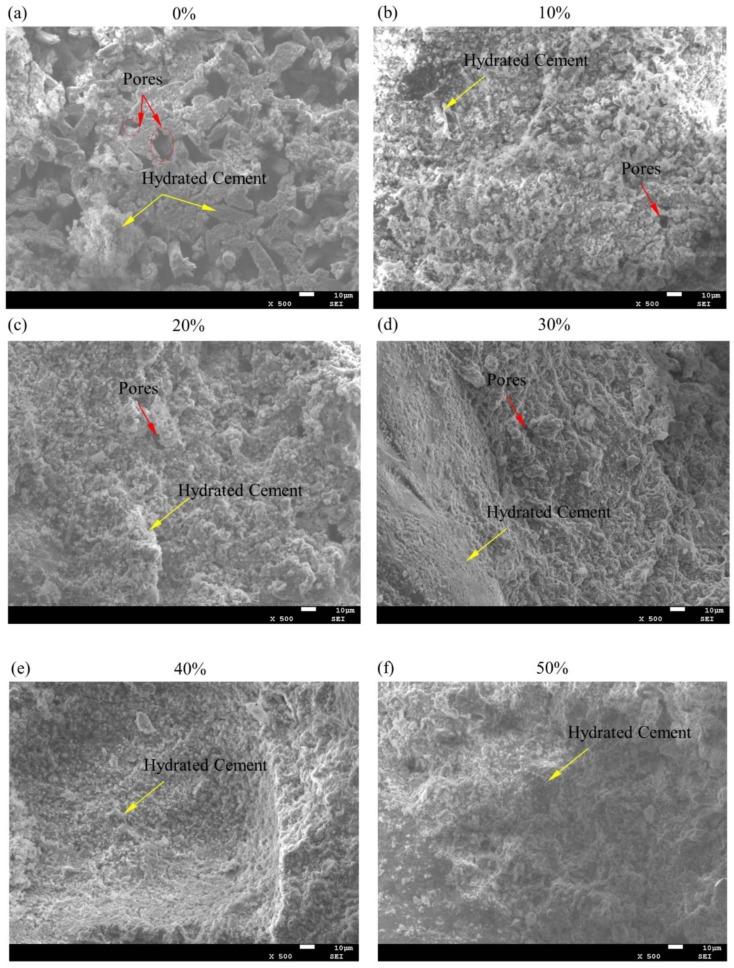

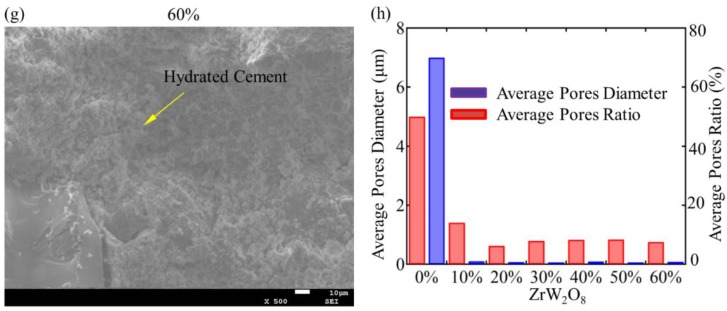
Scanning Electron Microscope (SEM) images of ZrW_2_O_8_ cementitious composites (ZCCs) of such percentages as 0% (**a**), 10% (**b**), 20% (**c**), 30% (**d**), 40% (**e**), 50% (**f**) and 60% (**g**), the size and ratio of the porosity of the ZCCs specimens in the SEM images (h). The ratios and sizes of porosities among those specimens decrease with the increase of ZrW_2_O_8_’s proportions.

In order to validate further macro-scale thermo-mechanical properties of ZCCs, the SEM imaging is conducted to amplify 500 times the surface of fragments of specimens, which is produced from flexural testing. SEM images of ZCCs of different percentages from 0% to 60% are listed in Figure 5a–g.

Comparison on ratios and sizes of pores imaged in those specimens as shown in Figure 5a–h implies that the greater the proportion of ZrW_2_O_8_ is, the lower the porous rate and size are. In regard to grain diameter of aggregates, the ISO sand is within 80–2000 µm, while ZrW_2_O_8_ is 4–7 µm. This is why 4–12 µm porous holes scatter in the section of pure cementitious composites, while fewer and smaller capillary voids occur in ZCCs regardless of the proportions. Actually, the ZrW_2_O8, as a type of filler, fills in the gaps of sand aggregates. When thermal load is exerted on the ZCCs or pure cementitious composite, ZrW_2_O_8_’s negative thermal expansion takes effect by virtue of C–S–H binding. Therefore, the existence of ZrW_2_O_8_ induces in ZCCs the reduction of the thermal expansion coefficient, however, as the proportion of ZrW_2_O_8_ rises, the TEC does not continuously decrease but fluctuates especially at 40%–60%. As for compressive and flexural strength, because ZrW_2_O_8_ only fills in 4–12 µm pore holes (nevertheless, loads are usually borne by more than 80 µm bonded sand aggregates), the mixed ZrW_2_O_8_ does not sharply shorten ZCCs’ strength, but keep stable on a 60%–70% strength of pure cementitious composite as the proportion increase until 60%. In a word, microstructures of ZCCs further confirm rationality of macro-scale mechanical testing.

## 4. Conclusions

In summary, to achieve cementitious composites insensitive to temperature variation, in order to avoid cracks and ensure structural integrity without such side effects as extra elastic modulus increase, extra hydration heat, and extra costs, we have demonstrated an approach to create ZrW_2_O_8_ cementitious composites with tunable thermal expansion coefficient ranging from 8.65 × 10^−6^ °C^−1^ to 2.48 × 10^−6^ °C^−1^. Through systematic macro-scale experiments on thermal expansion coefficient, compressive strength, and flexural strength, we have shown simultaneously that the thermal expansion coefficient of the cementitious composites can be tuned by varying the percentage of ZrW_2_O_8_ and the cementitious composites possess the effective strength to endure considerable loads. In particular, negative thermal expansion property of ZrW_2_O_8_ only plays a vatal role on compensating the positive thermal expansion when the ambient temperature is more than 80 °C. SEM imaging indicates that approximate 10 µm pores scatter in pure cementitious composites (0%), while fewer occur in 10–60% ZCCs, which further verify the macro-scale mechanical experimental results. The results presented here not only demonstrate the development of a new type of engineering cementitious composites with tunable thermal expansion but also offer a wide range of potential applications in civil engineering structures, where thermal stress induced cracks are of great concern in structural design. The findings provide us opportunities to extend the studies on ZCCs with longer curing ages, and from cementitious mortar (without coarse aggregate) on the small specimens to the concrete on the large specimens, to see how the materials developed can reduce thermal stress and maintain the structural integrity under extreme environmental conditions or 3D printing processes. Future work will be directed toward the real-world engineering applications of the newly developed cementitious composites. Importantly, for the measurement of TEC and thermal field in engineering structures, we will use the distributed optical fiber senor system. In addition, finite element-based computational models will be developed to predict the temperature field and thermal stress field. Collectively, the testing and computational modeling will provide us a better understanding on the effectiveness of the new developed cementitious composites in various engineering applications.

## Figures and Tables

**Table 1:** Mix design.

No.	ZrW_2_O_8_/Cement (%)	Cement/(g)	Sand/(g)	Water/(mL)	ZrW_2_O_8_/(g)
1	0	450	1350	225	0
2	10	450	1350	225	45
3	20	450	1350	225	90
4	30	450	1350	225	135
5	40	450	1350	225	180
6	50	450	1350	225	225
7	60	450	1350	225	270

## Nomenclature and Acronym

ZrW_2_O_8_	Zirconium tungstate
TEC	Thermal expansion coefficient
SEM	Scanning Electron Microscope
CCs	Cementitious Composites
MgO	Magnesium oxide
3D	Three dimensional
*σ*	Thermal stress
*E*	Young’s Modulus
*α*	TEC (Thermal expansion coefficient)
Δ*T*	Temperature increment
NTE	Negative thermal expansion
ZrV_2_O_7_	Zirconium pyrovanadate
ZCCs	ZrW_2_O_8_ cementitious composites
Z/C	ZrW_2_O_8_/Cement Ratio
*F_f_*	Force while the specimen is destroyed in the flexural test
[*σ*]*_f_*	Flexural strength
*L*	Distance between the supports in three-point flexural test
*b*	Width of cross section area.
$\alpha_{p}，\alpha_{s}，\alpha_{g}$	TECs of cement stone, sand and ZrW_2_O_8_, respectively
$E_{p}，E_{s}，E_{g}$	Elastic modulus of cement stone, sand and ZrW_2_O_8_
$V_{p}，V_{s}，V_{g}$	Volume fraction of cement stone, sand and ZrW_2_O_8_
